# Supplementary material for: Green Foaming of Biologically Extracted Chitin Hydrogels Using Supercritical Carbon Dioxide for Scaffolding of Human Osteoblasts
Source: Polymers (Basel). 2024 Jun 1;16(11):1569. doi: 10.3390/polym16111569 (PMC11174636; doi:10.3390/polym16111569)
Supplement: Supplementary file 1 [file polymers-16-01569-s001.zip › polymers-3015980-supplementary.pdf]

Table S1. Physical characteristics for S_A and S_M scaffolds after foaming with $scCO_2$ at 353 K and 175 bar.

MATERIAL	Mass (g)	Diameter (cm)	Thickness (mm)
S_A	4.28 ± 0.75[a]	4.65 ± 0.19[a]	1.23 ± 0.10[a]
S_M	1.09 ± 0.08[b]	3.63 ± 0.08[b]	1.24 ± 0.09[a]

The different letters in the columns mean that they are statistically different (Tukey- Kramer $p \leq 0.05$).

Table S2. Physical characteristics for S_M scaffolds after foaming with $scCO_2$ at different conditions of temperature and pressure.

CHARACTERISTICS	Temperature (K) at 175 bar			Pressure (bar) at 353 K			
	298	313	353	175	200	250	300
Mass (g)	0.87 ± 0.14[a]	0.91 ± 0.05[a]	0.51 ± 0.09[b]	0.52 ± 0.07[a]	0.42± 0.02[c]	0.45 ± 0.005[c]	0.48 ± 0.02[b]
Diameter (cm)	4.53 ± 0.08[a]	5.08 ± 0.12[a]	4.55 ± 0.05[a]	4.55 ± 0.05[a]	4.43 ± 0.05[b]	4.37 ± 0.08[b]	4.58 ± 0.10[a]
Thickness (mm)	1.10 ± 0.28[a]	1.28 ± 0.15[a]	1.21 ± 0.16[a]	1.21 ± 0.16[b]	1.19 ± 0.22[b]	1.36 ± 0.13[a]	1.46 ± 0.16[a]

The different letters in the rows mean that they are statistically different (Tukey- Kramer $p \leq 0.05$).

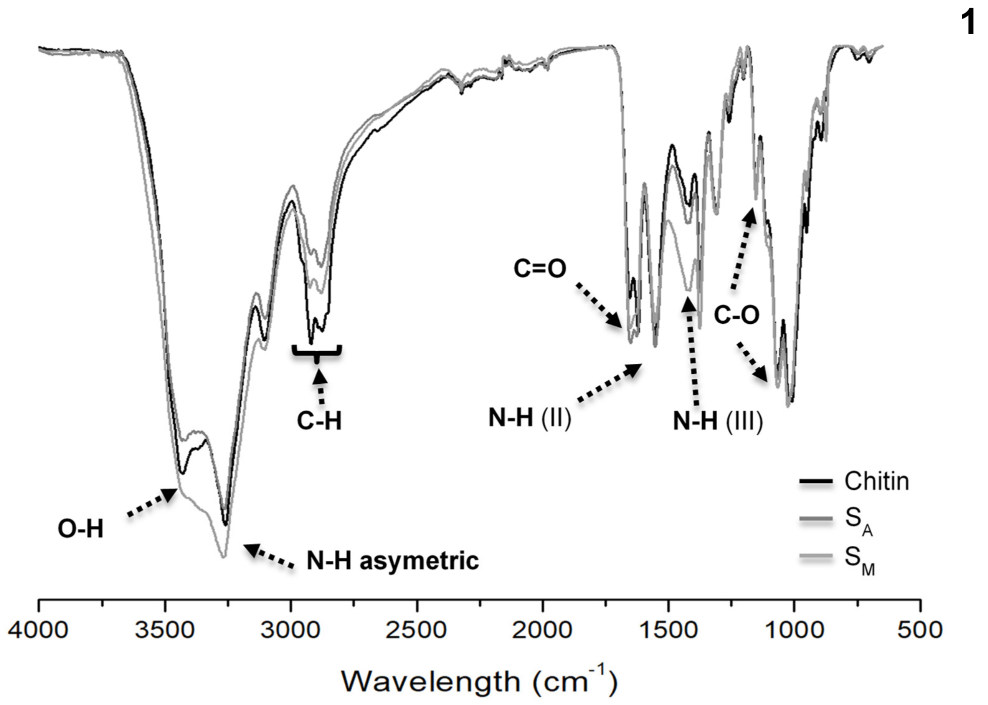
1
O-H
N-H asymetric
C-H
C=O
N-H (II)
N-H (III)
C-O
Chitin
S_A
S_M
4000 3500 3000 2500 2000 1500 1000 500
Wavelength (cm^{-1})

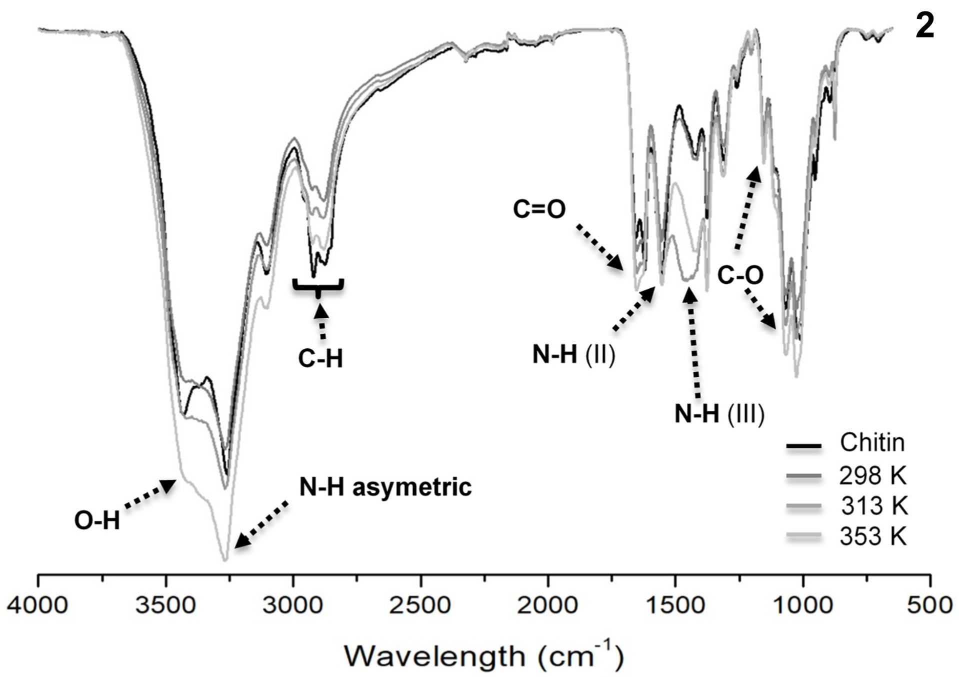
2
O-H
N-H asymetric
C-H
C=O
N-H (II)
N-H (III)
C-O
Chitin
298 K
313 K
353 K
4000 3500 3000 2500 2000 1500 1000 500
Wavelength (cm^{-1})

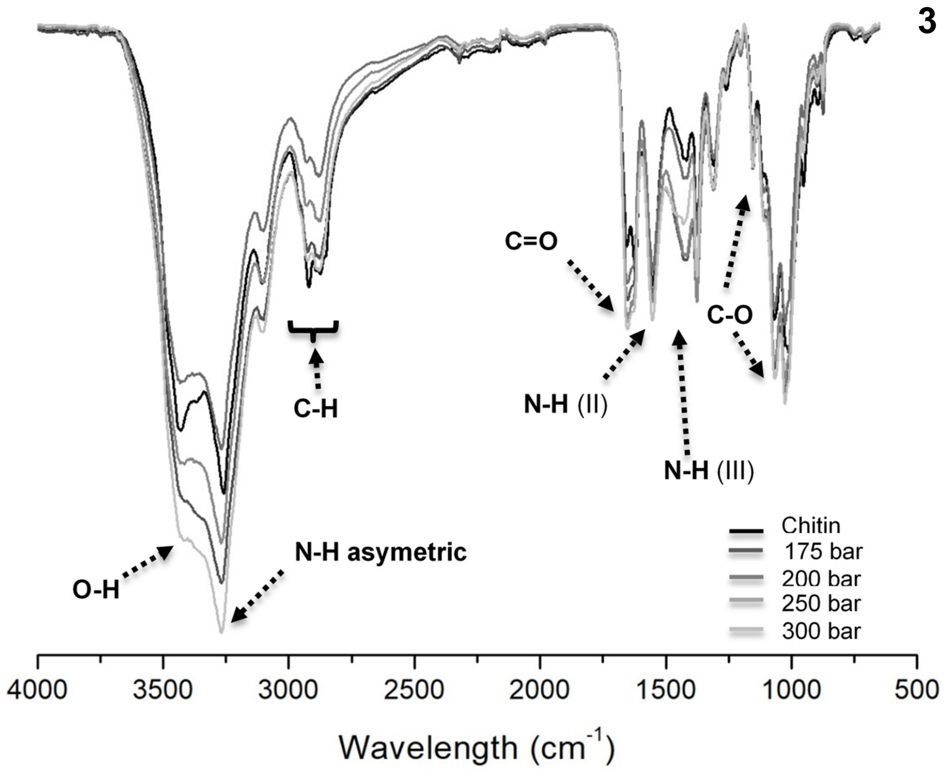

Figure S1. Infrared spectra of chitin and after treatment with $scCO_2$: S_A and S_M (1); S_M scaffolds varying temperatures at 175 bar (2) and varying pressures at 353 K (3).

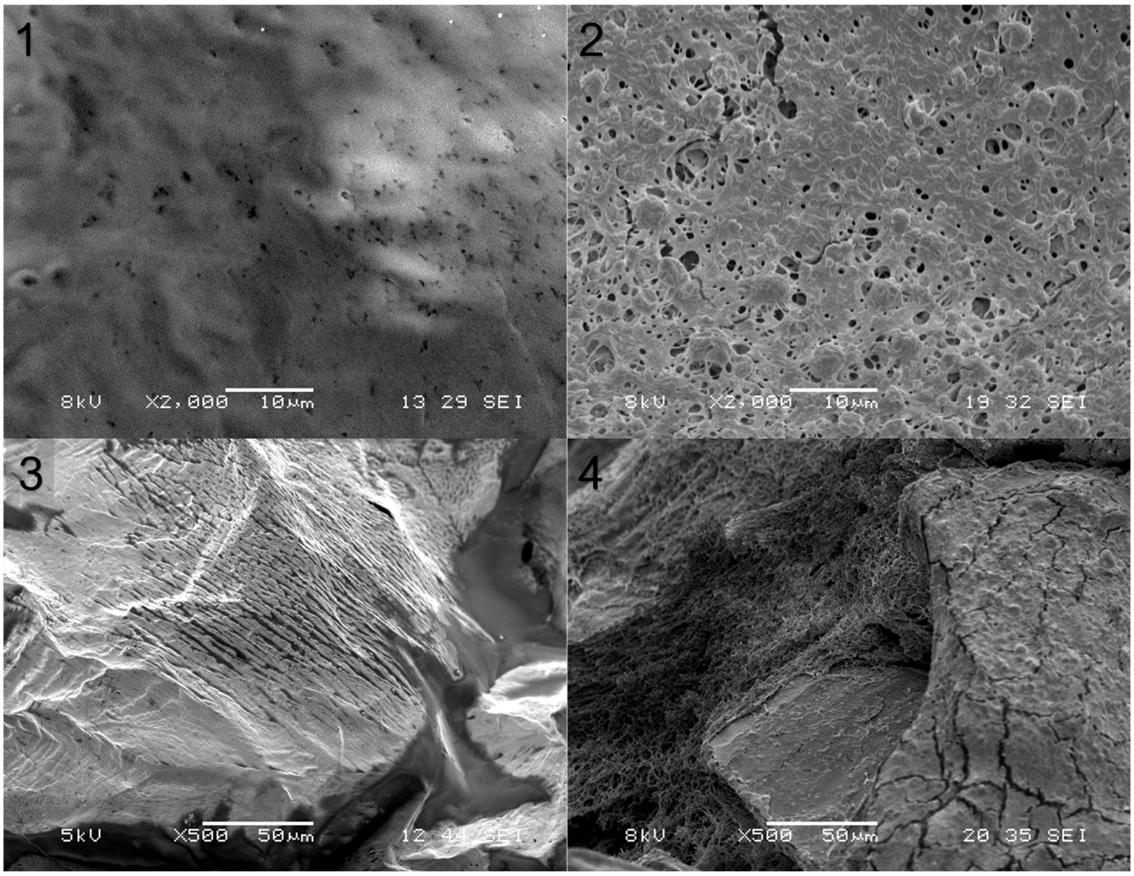

Figure S2. SEM micrographs of S_A (1: superficial, 3: transverse) and S_M (2 superficial, 4: transverse) SCF-processed porous materials at 353 K and 175 bar.

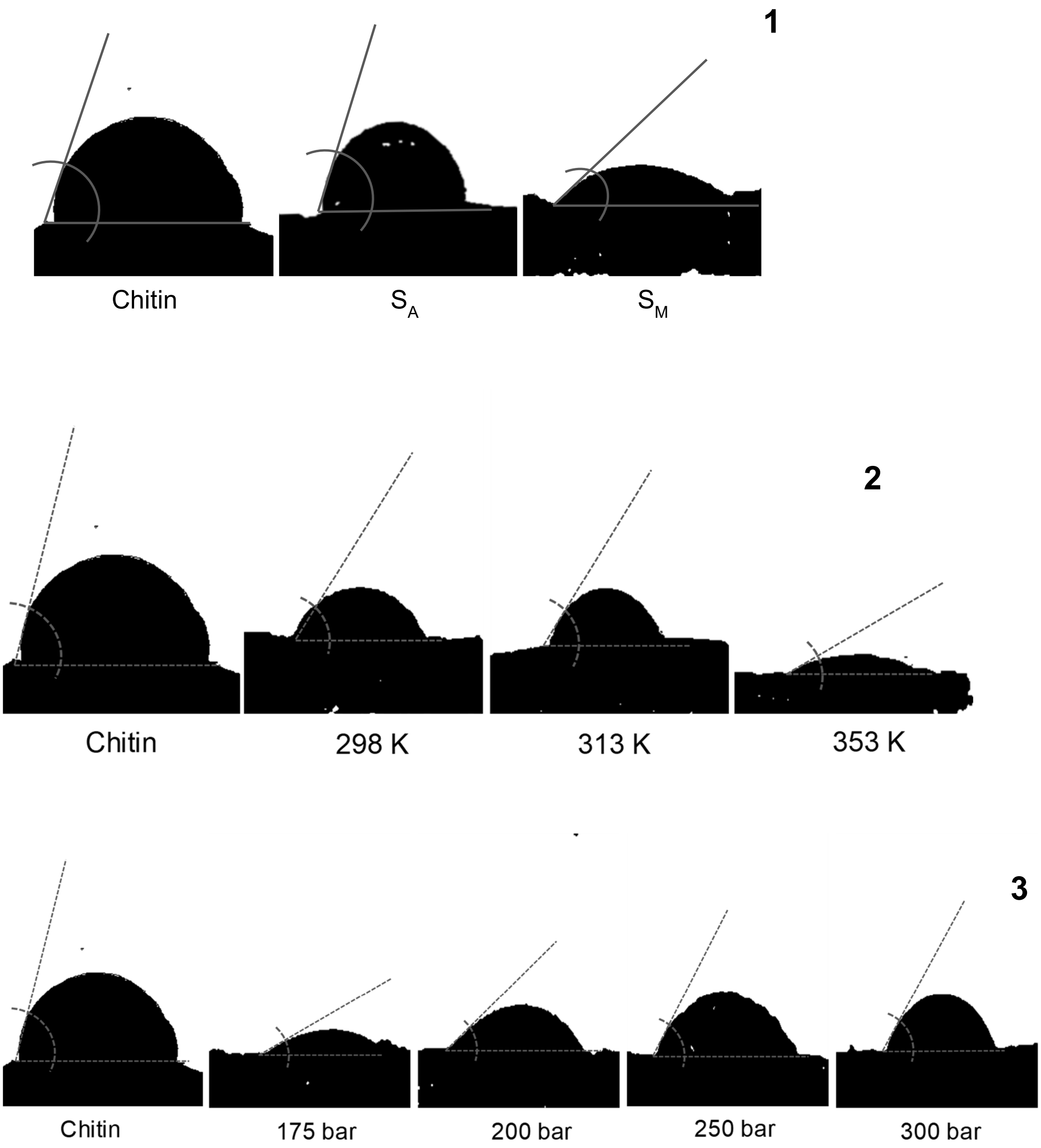

Figure S3. Determination of the contact angle of chitin and after treatment with $scCO_2$: S_A and S_M (1); S_M scaffolds varying temperatures at 175 bar (2) and varying pressures at 353 K (3).

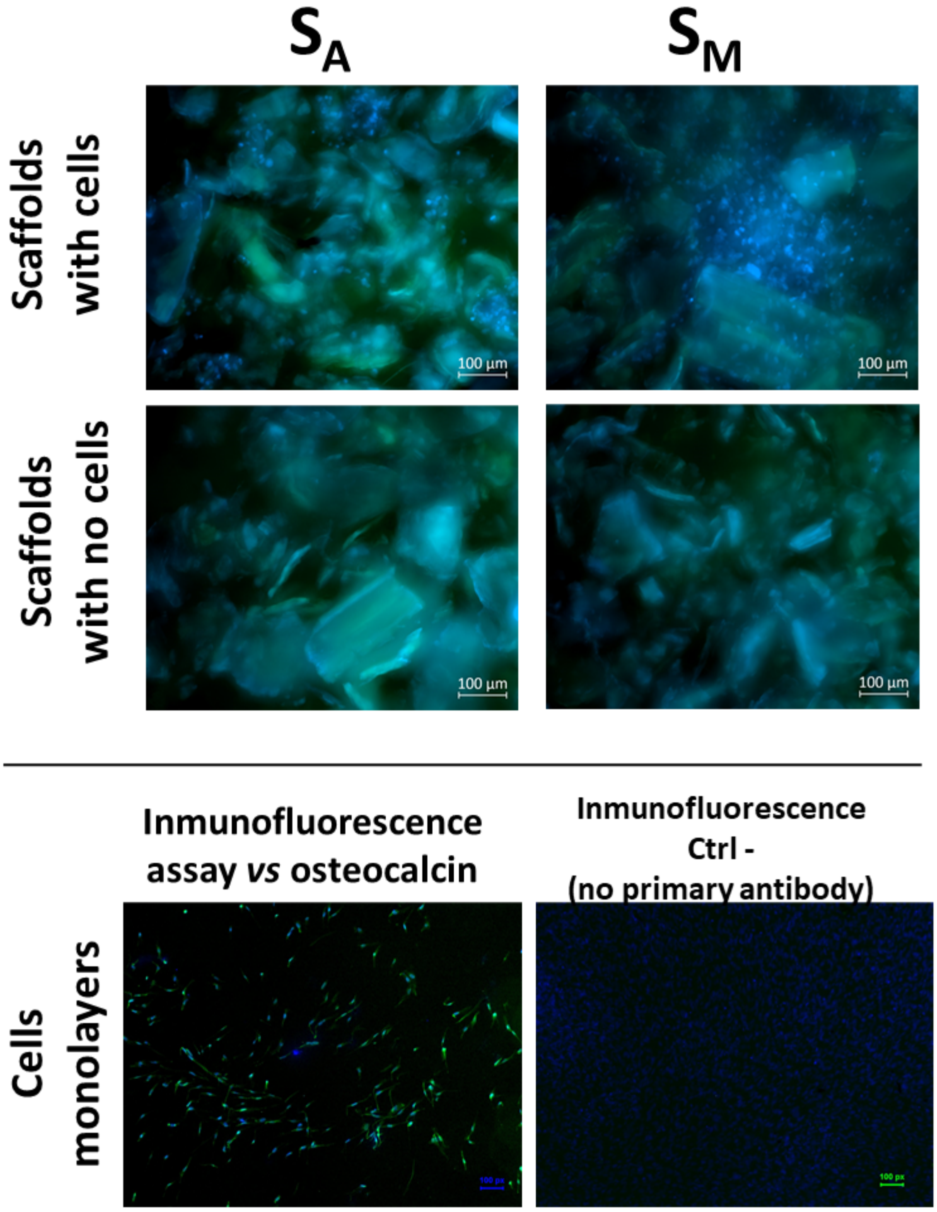

Figure S4. Representative micrographs of the imnnofluorescene assay performed on osteoblasts cultured for 14 days on the scaffolds. Scaffolds with no cells were also cultured for 14 days and used as controls to determine the autofluorescence of the scaffolds. Finally, osteoblasts monolayers were tested against osteocalcin immunofluorescence assay as positive controls of osteocalcin expression, and osteoblasts monolayers treated with no

primary antibody are presented as the Control negative (Ctrl-) of the technique to demonstrate the specificity of the primary antibody and the immunofluorescence technique used.
